# Vitamin K2 ameliorates pregnancy‐ and lactation‐associated osteopenia and postpartum depression

**DOI:** 10.1002/imt2.70180

**Published:** 2026-10-19

**Authors:** Wanqiong Yuan, Jing Hang, Jialin Jia, Jie Huang, Xuanqi Zheng, Huan Wang, Tong Wu, Hao Liu, Junxiong Zhu, Chenggui Zhang, Jinwen He, Yundi Zhang, Hong Wang, Xin Xiong, Na He, Haofan Bai, Jiamin Yan, Huijie Leng, Weili Zhu, Yingsheng Xu, Jie Shi, Chunli Song

**Affiliations:** ^1^ Department of Orthopedics Peking University Third Hospital Beijing China; ^2^ Engineering Research Center of Bone and Joint Precision Medicine, Ministry of Education Beijing China; ^3^ Beijing Key Laboratory of Advanced Bioadaptable Orthopedic Implants Beijing China; ^4^ State Key Laboratory of Female Fertility Promotion, Center for Reproductive Medicine, Department of Obstetrics and Gynecology Peking University Third Hospital Beijing China; ^5^ National Clinical Research Center for Obstetrics and Gynecology, Key Laboratory of Assisted Reproduction, Ministry of Education Beijing Key Laboratory of Reproductive Endocrinology and Assisted Reproduction Beijing China; ^6^ Beijing Advanced Center of Cellular Homeostasis and Aging‐Related Diseases, Institute of Advanced Clinical Medicine Peking University Beijing China; ^7^ Department of Orthopedics, Beijing Chaoyang Hospital Capital Medical University Beijing China; ^8^ Department of Orthopedics Surgery, The Second Affiliated Hospital Zhejiang University School of Medicine Hangzhou China; ^9^ Department of Orthopaedics Shandong Provincial Hospital Affiliated to Shandong First Medical University Jinan China; ^10^ Department of Pharmacy Peking University Third Hospital Beijing China; ^11^ School of Basic Medical Sciences Peking University Beijing China; ^12^ National Institute on Drug Dependence and Beijing Key Laboratory of Drug Dependence Research Peking University Beijing China; ^13^ Department of Neurology Peking University Third Hospital Beijing China

## Abstract

Vitamin K2 (VitK2) supplementation ameliorates comorbid pregnancy‐ and lactation‐associated osteopenia (PLO) and postpartum depression (PPD). These improvements are accompanied by reduced relative abundance of *Bacteroides uniformis* (*B*. *uniformis*) and lower serum deoxycholic acid (DCA) levels. DCA inhibits osteoblast differentiation, promotes osteoclast differentiation, and contributes to an imbalance between hippocampal glutamatergic and GABAergic signaling. Maternal VitK2 supplementation also improves offspring cognition. These findings suggest that modulation of the gut microbiota–bile acid axis may contribute to the beneficial effects of VitK2, supporting the potential of VitK2 as a therapeutic agent for PLO and PPD.

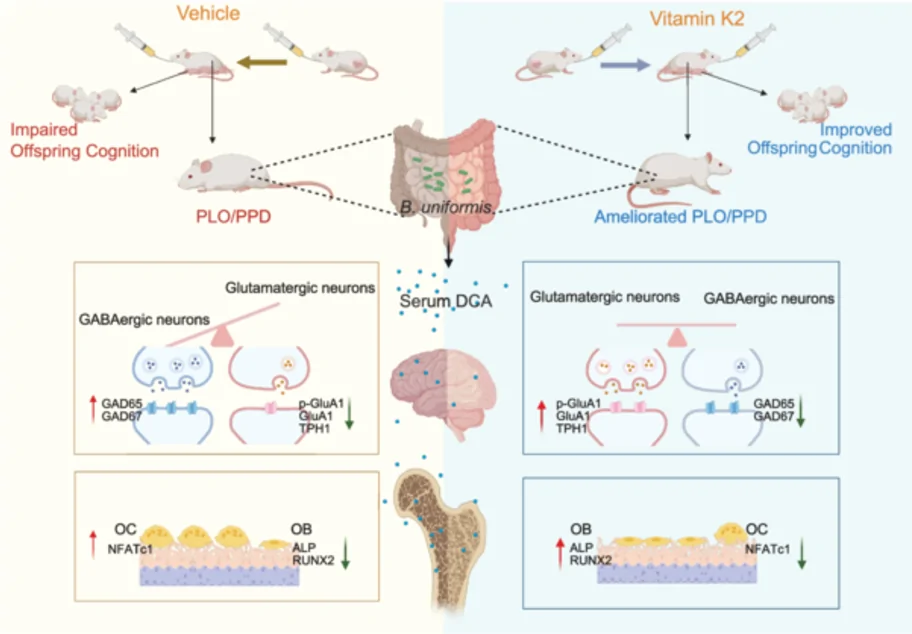

To the Editor,

Pregnancy‐ and lactation‐associated osteopenia (PLO) and postpartum depression (PPD) co‐occur clinically, suggesting shared etiological factors and pathogenic mechanisms [1]. PLO is a severe early‐onset osteopenia, and bone deficits often persist after weaning [2]. Simultaneously, perinatal mental health conditions pose a substantial burden, particularly in low‐ and middle‐income countries, inflicting severe adverse effects on maternal well‐being and offspring neurodevelopment [3, 4]. However, treatment options during pregnancy and lactation are constrained by limited safety evidence and drug‐specific risks, leaving a significant therapeutic gap for managing this comorbidity.

The gut microbiota undergoes dramatic remodeling during pregnancy and lactation [5]. Gut microbial homeostasis is key to the maintenance of a successful pregnancy [6]. It is well established that the microbiota profoundly modulates bone metabolism and neurohomeostasis, largely through circulating metabolites such as bile acids [7, 8, 9]. Consequently, the gut microbiota emerges as a highly promising candidate mediator that plays a crucial role in the pathogenesis of PLO and PPD comorbidity.

Vitamin K2 (VitK2) serves as an essential multifunctional nutrient in human physiology, playing a role in various conditions, including the dysregulation of bone homeostasis and neurological disorders [10, 11]. Furthermore, it has been approved as a dietary supplement in many countries and recognized as a treatment for osteoporosis clinically [12]. Because VitK2 plays an important role in pregnant women and infants [13, 14], it presents a potentially safe therapeutic option during pregnancy and lactation. In this study, we investigated whether VitK2 could bridge this therapeutic gap by modulating the gut‐brain and gut‐bone axes.

## VITK2 SUPPLEMENTATION ALLEVIATES PLO AND PPD PHENOTYPES

To recapitulate the phenotype of PLO, female rats were maintained on a low‐calcium diet throughout gestation and lactation (Figure S1A and Table S1). Consistent with effective calcium deprivation, maternal serum calcium levels were significantly depleted (Figure S1B). Micro‐computed tomography (micro‐CT) analysis revealed pronounced bone loss in the low‐calcium diet group (Figure S1C–G). Consistently, bone mineral density (BMD) and mechanical strength were significantly decreased (Figure S1H–K). Hereafter, the low‐calcium diet group is referred to as the “PLO” group.

Serum VitK2 naturally declines during normal pregnancy, lactation, and calcium restriction (Figure S2A,B). As shown in the treatment protocol (Figure 1A), exogenous VitK2 supplementation dose‐dependently increased serum VitK2, but PLO rats paradoxically exhibited elevated serum VitK2 at both Days 21 and 42 (Figure 1B). Given that gut microbes play important roles in converting vitamin K1 (VitK1) to VitK2 [15], we assessed VitK1 dynamics. VitK1 levels initially declined by Day 21 but re‐accumulated by Day 42 (Figure S2C,D), suggesting that VitK1 serves as a mobilizable reservoir that supports compensatory VitK2 biosynthesis both after lactation and under a calcium‐deficient diet. Furthermore, exogenous VitK2 supplementation reduced endogenous VitK1 (Figure S2E), indicating that adequate intake may reduce the need for microbial conversion.

**Figure 1:**
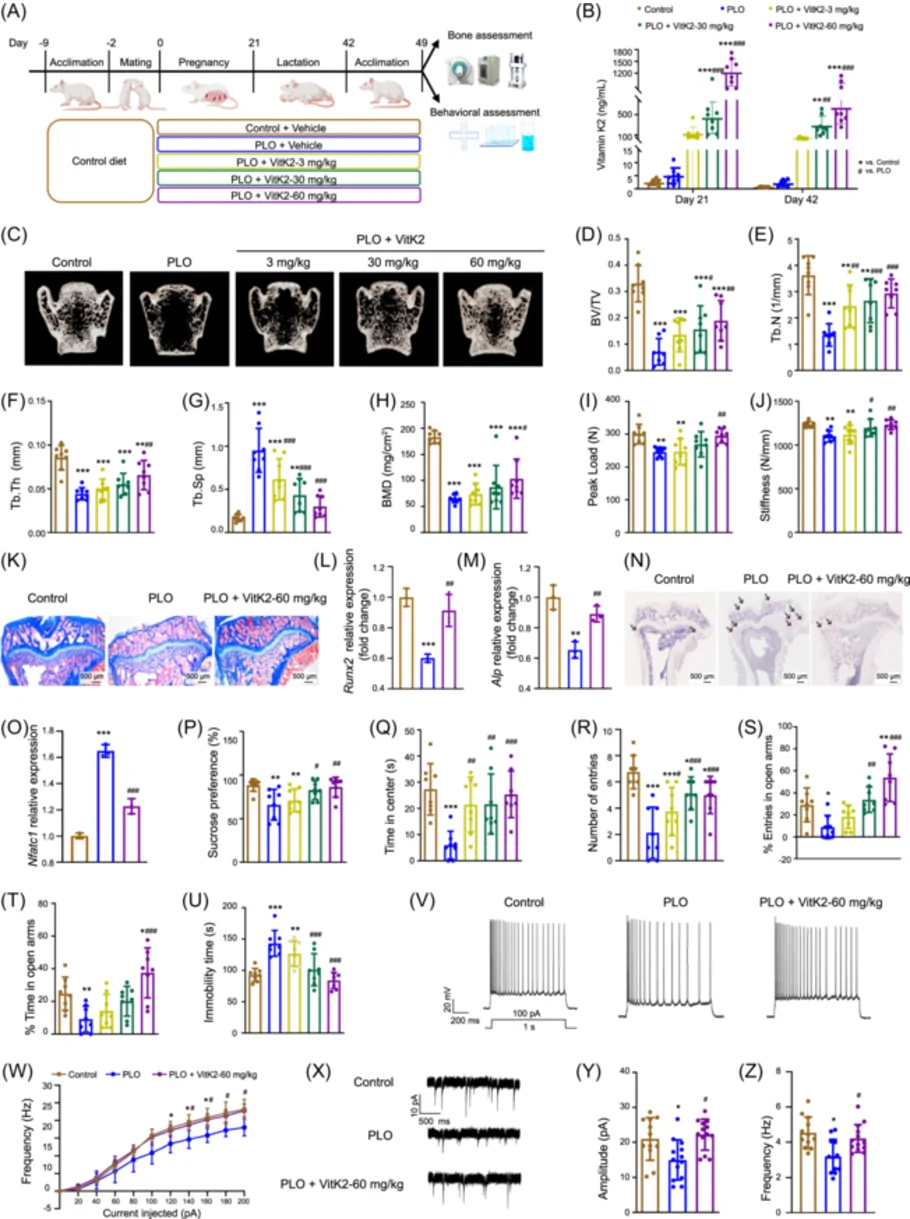
Vitamin K2 (VitK2) ameliorated pregnancy‐ and lactation‐associated osteopenia (PLO) and postpartum depression (PPD) in maternal rats. (A) Schematic illustration of the treatment protocol. (B) Serum VitK2 levels in rats. (C) Representative micro‐computed tomography (micro‐CT) images of the fourth lumbar (L4) vertebra. Quantitative micro‐CT analysis of L4 vertebral parameters: bone volume/tissue volume (BV/TV) (D), trabecular number (Tb.N) (E), trabecular thickness (Tb.Th) (F), and trabecular separation (Tb.Sp) (G). (H) Bone mineral density (BMD) of the lumbar spine was analyzed by dual‐energy X‐ray absorptiometry. Biomechanical properties, including peak load (I) and stiffness (J) of the lumbar spine were analyzed by MTS Landmark Systems. (K) Representative Masson's trichrome staining of the tibia (scale bar: 500 μm). (L) The expression of runt‐related transcription factor 2 (*Runx2*) in bone measured by real‐time PCR. (M) The expression of alkaline phosphatase (*Alp*) in bone measured by real‐time PCR. (N) Representative tartrate‐resistant acid phosphatase (TRAP) staining of the tibia (scale bar: 500 μm). (O) The expression of nuclear factor of activated T cells, cytoplasmic 1 (*Nfatc1*) in bone by real‐time PCR. Behavioral assessments of maternal rats post‐lactation: Sucrose preference test (P); Open field test showing time spent in the center (Q) and number of entries (R); Elevated plus maze showing percentage of entries in open arms (S) and percentage of time spent in open arms (T); Forced swim test (U). The above bone indices and depression‐related behavioral measures were all corrected for body weight. (V) Representative traces of action potentials evoked by 100 pA current injection in hippocampal neurons. (W) Frequency of action potentials. (X) Representative traces of spontaneous excitatory postsynaptic currents (sEPSCs) in hippocampal cornu ammonis 1 (CA1) pyramidal neurons. (Y) Mean peak amplitude of sEPSCs. (Z) Mean frequency of sEPSCs. For panels (B–K), (N), and (P–U), *n* = 8 rats per group. For panels (L), (M), and (O), real‐time PCR data represent statistical values from three independent biological replicates (*n* = 3 per group). For action potential recordings (panels V and W), 12–13 cells from *n* = 4–5 rats per group. For sEPSC recordings (panels X–Z), 11–12 cells from n = 4 rats per group. * or ^#^ *p* < 0.05, ** or ^##^ *p* < 0.01, *** or ^###^ *p* < 0.001.

Next, we evaluated the therapeutic efficacy of VitK2 against PLO and its comorbid phenotypes. We observed that body weights of the maternal rats did not differ significantly among groups 1 week after offspring weaning (Figure S3A). Although maternal serum calcium remained low in VitK2‐supplemented PLO rats (Figure S3B), VitK2 treatment at 60 mg/kg increased serum parathyroid hormone levels and reduced serum parathyroid hormone‐related peptide levels relative to the PLO group (Figure S3C,D). VitK2 exerted a potent osteoprotective effect by restoring trabecular microarchitecture (Figure 1C–G, Figure S3E), increasing BMD and mechanical strength in a dose‐dependent manner in PLO rats, with the 60 mg/kg dose producing the greatest overall improvement in the assessed bone parameters (Figure 1H–J, Figure S3F); this dose was therefore selected for subsequent assays. Furthermore, VitK2 promoted bone formation with upregulating osteogenic markers (Figure 1K–M), and inhibited bone resorption with reducing the aberrant promotion in osteoclast differentiation (Figure 1N–O).

In addition, PLO rats exhibited obvious postpartum depressive symptoms, which were reversed by VitK2 supplementation in a dose‐dependent manner, with the 60 mg/kg dose exhibiting the most potent antidepressant efficacy (Figure 1P–Z, Figure S4) and ameliorating behavioral deficits, as evidenced by the increased sucrose preference (Figure 1P), increased exploratory activity in both the open field test (Figure 1Q,R) and the elevated plus maze (Figure 1S,T), and reduced immobility in the forced swim test (Figure 1U). Electrophysiological experiments further corroborated these behavioral findings. The action potential firing rate was reduced in the PLO group, an effect that was rescued by VitK2 supplementation (Figure 1V,W). Moreover, VitK2 increased spontaneous excitatory postsynaptic currents (sEPSCs) amplitude and frequency, which decreased in the PLO group (Figure 1X–Z). Taken together, these data not only validate the comorbidity between PLO and PPD but also highlight the dual therapeutic efficacy of VitK2 against these co‐occurring disorders.

## VITK2 SUPPRESSES THE PATHOLOGICAL EXPANSION OF *BACTEROIDES UNIFORMIS* (*B*. *UNIFORMIS*)

To explore the underlying mechanisms, we first measured serum corticosterone levels and found that VitK2 failed to reverse the corticosterone reduction observed in the PLO group (Figure S5). Additionally, exogenous osteocalcin (OCN) supplementation did not ameliorate PLO and PPD, whereas VitK2 supplementation remained effective in improving bone mass and depressive phenotypes in *Ocn* knockout (KO) mice (Figure S6). These findings suggest that the benefits of VitK2 do not require OCN.

Given that gut microbial homeostasis is inextricably linked to both bone and neurological health, we performed 16S ribosomal RNA (rRNA) and metagenomic sequencing. Global analyses revealed alterations in microbial diversity in PLO rats, which were partially normalized by VitK2 supplementation (Figure S7A–D). Kyoto Encyclopedia of Genes and Genomes (KEGG) pathway analysis highlighted enrichments in metabolism‐related biological processes (Figure S7E). Taxonomically, the PLO group was characterized by a marked expansion of the family *Bacteroidaceae* and the genus *Bacteroides* by linear discriminant analysis effect size (LEfSe) analysis (Figure 2A) and heatmap analyses (Figure S7F,G). Random forest analysis revealed that *Bacteroides* was the most important variable distinguishing the groups (Figure S7H). VitK2 supplementation (especially 60 mg/kg) reduced the elevated relative abundance of *Bacteroidaceae* and *Bacteroides* in the PLO group (Figure 2B, Figure S7I). *Bacteroides* are known to produce menaquinones (MKs) [16]; we therefore speculate that sufficient exogenous VitK2 supply fulfilled the physiological requirement, alleviating the selective pressure for their compensatory overgrowth under calcium stress. These data suggest a potential role of *Bacteroides* in the treatment of PLO and PPD by VitK2. Correlation analysis revealed a strong association between *Bacteroidaceae*/*Bacteroides* abundance, deteriorated bone parameters, and depressive phenotypes (Figure S7J,K), implicating this microbial shift in disease pathogenesis.

**Figure 2:**
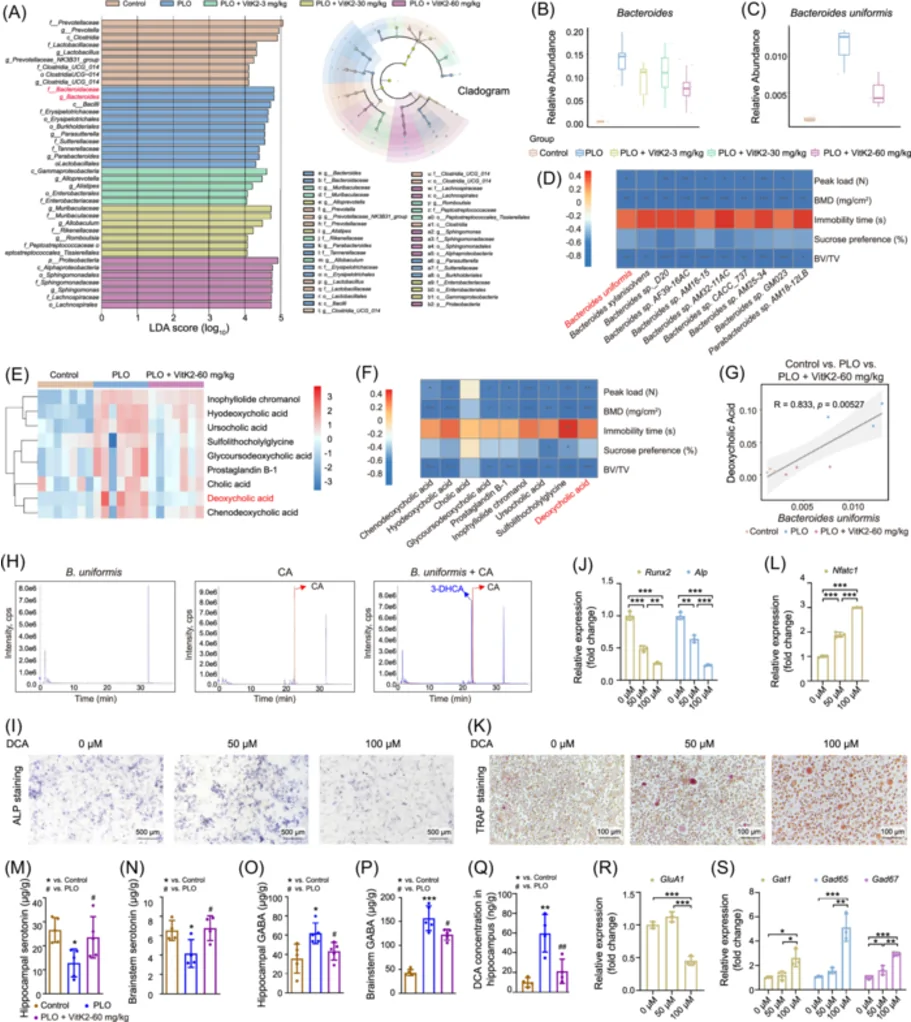
*Bacteroides uniformis* (*B*. *uniformis*)‐deoxycholic acid (DCA) axis regulates bone remodeling and neurotransmission. (A) 16S ribosomal RNA (rRNA) sequencing linear discriminant analysis effect size (LEfSe) analysis identifying differentially abundant taxa. (B) Relative abundance of the genus *Bacteroides*. (C) Relative abundance of the species *Bacteroides uniformis* (*B. uniformis*). (D) Spearman's correlation analysis between bone/depressive‐like behavioral indices and species. (E) Heatmaps of metabolites exhibiting an inverted V‐shaped trend in abundance by serum metabolomics. (F) Correlation analysis between the inverted V‐shaped trend metabolites and bone/depressive‐like behavioral indices. (G) Correlation analysis between *B. uniformis* and DCA. (H) Mass spectrometry detection of bile acid biotransformation by *B. uniformis*. (Left) supernatant from *B. uniformis*‐only culture; (Middle) supernatant from cholic acid (CA)‐only culture; (Right) supernatant from *B. uniformis* cultured with CA. Red arrows indicate the peak of substrate CA, and blue arrows indicate the product peak of 3‐dehydrocholic acid (3‐DHCA). (I) ALP staining of MC3T3‐E1 cells treated with different concentrations of DCA (scale bar: 500 μm). (J) The relative expression of *Runx2* and *Alp* analyzed by real‐time PCR in MC3T3‐E1 cells treated with different concentrations of DCA in osteoblast differentiation medium. (K) TRAP staining of RAW264.7 cells treated with DCA (scale bar: 100 μm). (L) The relative expression of *Nfatc1* was analyzed by real‐time PCR in RAW264.7 cells treated with different concentrations of DCA in osteoclast differentiation medium. (M and N) High‐performance liquid chromatography (HPLC) quantification of serotonin levels in the hippocampus (M) and brainstem (N), respectively. (O and P) HPLC quantification of γ‐aminobutyric acid (GABA) levels in the hippocampus (O) and brainstem (P), respectively. (Q) Concentration of DCA in the hippocampus by HPLC. (R) The expression of *GluA1* in glutamatergic neurons differentiated from SH‐SY5Y cells treated with different concentrations of DCA by real‐time PCR. (S) The expression of *GABA transporter 1* (*Gat1*), glutamate decarboxylase (*Gad*)*65*, and *Gad67* in GABAergic neurons differentiated from SH‐SY5Y cells treated with different concentrations of DCA by real‐time PCR. Real‐time PCR data represent statistical values from three independent replicate experiments. * or ^#^ *p* < 0.05, ** or ^##^ *p* < 0.01, ***p < 0.001.

Metagenomic sequencing further identified *B. uniformis* as the most abundant species within the genus *Bacteroides* in feces (Figure S8A–D). The abundance of *B. uniformis* increased in the PLO group but was suppressed by VitK2 supplementation (Figure 2C). The correlations between the differentially abundant species and disease parameters suggested that the abrogation of abnormally increased *B. uniformis* by VitK2 may contribute to the amelioration of PLO and PPD (Figure 2D). These results suggest that severe calcium stress may induce a compensatory feedback mechanism driving *B. uniformis* proliferation to elevate endogenous VitK2. Providing sufficient exogenous VitK2 eliminates this physiological requirement, thereby normalizing the abundance of *B. uniformis*.

## *B*. *UNIFORMIS*‐ASSOCIATED DEOXYCHOLIC ACID (DCA) MEDIATES VITK2‐DEPENDENT PROTECTION AGAINST PLO AND PPD

Serum metabolomics identified primary and secondary bile acids as key disease‐correlated metabolites (Figure S9A‐H), while functional analysis of metagenomic sequencing revealed enrichment of secondary bile acid biosynthesis (Figure S8E). Specifically, DCA accumulated abnormally in the PLO group but was significantly reduced by VitK2 supplementation (Figure 2E). DCA levels strongly correlated with bone and depressive indices, as well as *B. uniformis* abundance (Figure 2F,G, Figure S9I–K). We found that *B. uniformis* actively converts cholic acid into 3‐dehydrocholic acid, a critical DCA intermediate (Figure 2H), whereas *Clostridium scindens*, which completes DCA synthesis, showed no statistically significant difference in the relative abundance among groups (Figure S8F).

Multi‐omics analysis linked the amelioration of PLO by VitK2 to mineral absorption pathways (Figure S9H,L). Investigating the direct effects of DCA on bone cells revealed that high DCA concentrations suppressed osteoblast differentiation and downregulated runt‐related transcription factor 2 (*Runx2*) and alkaline phosphatase (*Alp*) expression (Figure 2I,J), while concurrently promoting osteoclastogenesis and upregulating nuclear factor of activated T cells, cytoplasmic 1 (*Nfatc1*) expression (Figure 2K,L).

To elucidate the neural mechanisms, multi‐omics and metagenomic profiling highlighted neuroactive ligand‐receptor interactions, alongside glutamatergic, serotonergic, and γ‐aminobutyric acid (GABA)‐mediated synapses as key targets (Figures S9H,L, S10A–C). PLO rats exhibited increased glutaminase and glutamate decarboxylase (GAD) abundance (Figure S10D). Moreover, a widespread neurochemical disturbance was marked by decreased serotonin, acetylcholine, and taurine, alongside elevated GABA in the brain. VitK2 partially reversed these neurotransmitters (Figure 2M–P, Figure S10E,F), restored tryptophan hydroxylase and GAD expression (Figure S10G,H), and rescued the activation of hippocampal glutamate receptor 1 (GluA1), a subunit of α‐amino‐3‐hydroxy‐5‐methyl‐4‐isoxazolepropionic acid (AMPA) receptors (Figure S10I), which tightly correlated with behavioral improvements (Figure S10J). Furthermore, pathologically elevated hippocampal DCA in PLO rats was normalized by VitK2 (Figure 2Q). In vitro, DCA (100 μM) exposure shifted SH‐SY5Y cell differentiation away from glutamatergic and toward GABAergic neurons, directly disrupting the excitatory‐inhibitory equilibrium essential for mental health (Figure 2R,S). Notably, DCA did not affect cell viability at these concentrations (Figure S10K).

## MATERNAL SUPPLEMENTATION OF VITK2 IMPROVES OFFSPRING COGNITION WITHOUT AFFECTING SKELETAL DEVELOPMENT

Beyond the maternal benefits, we evaluated the offspring impact of VitK2. Maternal VitK2 supplementation significantly ameliorated cognitive impairment in both male and female offspring. Concurrently, immunohistochemical analysis demonstrated a marked increase in doublecortin (DCX)‐positive neurons in the hippocampus. Notably, these neurodevelopmental improvements occurred without affecting their physiological skeletal growth (Figures S11, S12).

## DISCUSSION AND PROSPECTS

While most research focuses on postmenopausal osteoporosis, the present study highlights PLO and demonstrates that VitK2 effectively treats both PLO and its comorbid PPD. Mechanistically, our findings suggest that the benefits of VitK2 do not require OCN (Figure S6). Furthermore, PLO rats exhibited a paradoxical elevation in endogenous VitK2, which should theoretically enhance the activation of classic vitamin K‐dependent proteins like matrix γ‐carboxyglutamic acid (Gla) protein and growth arrest‐specific protein 6 (Gas6). However, this compensatory increase failed to rescue the osteopenic or depressive phenotypes. Thus, other canonical vitamin K‐dependent proteins are unlikely to be the primary mediators. Instead, our data demonstrate that VitK2 exerts its therapeutic benefits by modulating the *B. uniformis*‐DCA axis.

Regarding offspring benefits, we observed that offspring serum VitK2 levels increased following high‐dose maternal supplementation. However, supplementation spanned both pregnancy and lactation, preventing us from distinguishing placental transfer, milk transfer, and indirect maternal effects. Several hypotheses concerning placental VitK2 handling warrant investigation. First, passive diffusion across placental membranes could contribute to VitK2 transfer, although its extent remains uncertain. Second, lipoprotein‐bound VitK2 may traverse the syncytiotrophoblast through mechanisms similar to transplacental vitamin A delivery [17], possibly involving lipoprotein processing pathways. Third, the placenta may act as a specific physiological reservoir that actively accumulates VitK2 and releases it slowly, thereby protecting the fetus from acute supraphysiological spikes [18]. Finally, placenta‐derived extracellular vesicles, which traverse both the placental and blood‐brain barriers to directly influence fetal neurodevelopment [19], provide a promising though currently speculative framework for exosomal VitK2 delivery that warrants future investigation.

Furthermore, the neuroprotective potential of VitK2 may extend beyond the perinatal period, as it successfully ameliorated low‐calcium diet‐induced depression‐like phenotypes in nulliparous mice (Figure S13). These findings highlight the need to explore its efficacy in other models of depression and broader neurological disorders.

Finally, our experimental design evaluated three dose levels and revealed that the therapeutic efficacy of VitK2 against PLO and PPD is dose‐dependent. Regarding clinical feasibility, menaquinone‐4 (MK‐4) at 45 mg/day has been approved for treating postmenopausal osteoporosis [12], demonstrating an excellent long‐term safety profile. However, extensive clinical trial data for MK‐4 supplementation during gestation and lactation are currently lacking. These findings highlight the need for rigorous clinical evaluation to establish safe and effective dosing guidelines for peripartum women.

In conclusion, our study demonstrates that VitK2 supplementation during pregnancy and lactation effectively ameliorates PLO and PPD. Maternal VitK2 supplementation also rescued offspring cognitive impairment and enhanced hippocampal neurogenesis. By modulating gut dysbiosis and bile acid metabolism, VitK2 serves as an effective and promising agent to simultaneously protect maternal skeletal integrity and mental health while conferring developmental benefits to the offspring.

## AUTHOR CONTRIBUTIONS

**Wanqiong Yuan**: Methodology; investigation; data curation; writing—review and editing; writing—original draft; funding acquisition. **Jing Hang**: Methodology; writing—original draft; writing—review and editing. **Jialin Jia**: Methodology; investigation. **Jie Huang**: Investigation. **Xuanqi Zheng**: Investigation. **Huan Wang**: Investigation. **Tong Wu**: Investigation. **Hao Liu**: Investigation. **Junxiong Zhu**: Investigation. **Chenggui Zhang**: Investigation. **Jinwen He**: Investigation. **Yundi Zhang**: Investigation. **Hong Wang**: Investigation. **Xin Xiong**: Methodology. **Na He**: Investigation. **Haofan Bai**: Investigation. **Jiamin Yan**: Writing—original draft. **Huijie Leng**: Investigation. **Weili Zhu**: Funding acquisition; writing—original draft. **Yingsheng Xu**: Supervision; writing—original draft. **Jie Shi**: Supervision; funding acquisition; writing—original draft. **Chunli Song**: Conceptualization; supervision; funding acquisition; writing—review and editing.

## CONFLICT OF INTEREST STATEMENT

The authors declare no conflicts of interest.

## ETHICS STATEMENT

The ethical approval (No. LA2021553) for all animal experiments in this study was obtained from the Ethics Committee of Biomedical Science of Peking University.

## Supporting information


**Figure S1:** Low‐calcium diet induces pregnancy‐ and lactation‐associated osteopenia (PLO) in maternal rats.
**Figure S2:** Serum vitamin K levels under different physiological and dietary conditions.
**Figure S3:** Effects of VitK2 on calcium‐regulating hormones and bone properties in PLO rats.
**Figure S4:** Maternal serum serotonin levels during pregnancy and lactation.
**Figure S5:** Serum corticosterone levels.
**Figure S6:** VitK2 ameliorated low‐calcium diet‐induced PLO and PPD independently of osteocalcin (OCN).
**Figure S7:** 16S rRNA gene sequencing analysis of the maternal rats.
**Figure S8:** Metagenomic profiling of the gut microbiota in maternal rats.
**Figure S9:** Multi‐omics analysis of the maternal rats.
**Figure S10:** Multi‐omics sequencing reveals that VitK2 ameliorates postpartum depression.
**Figure S11:** Maternal VitK2 supplementation improves female offspring cognition.
**Figure S12:** Maternal VitK2 supplementation improves cognitive function in male offspring.
**Figure S13:** VitK2 alleviates depressive‐like behaviors in nulliparous mice fed with a low‐calcium diet.

**Table S1:** Composition of the experimental diets.
**Table S2:** Primer sequences for real‐time PCR.

## Data Availability

The 16S rRNA sequencing data used in this study are deposited in the Genome Sequence Archive (GSA) at the National Genomics Data Center (NGDC) under the accession CRA048927 (https://ngdc.cncb.ac.cn/gsa/browse/CRA048927). The metagenomic sequencing data are deposited in the GSA under the accession CRA048577 (https://ngdc.cncb.ac.cn/gsa/browse/CRA048577). The serum metabolomic data are publicly available from the OMIX database under the accession OMIX019671 (https://ngdc.cncb.ac.cn/omix/release/OMIX019671). The data and scripts used to generate the figures in this study can be found on GitHub: https://github.com/yuanwanqiong/Vitamin-K2-ameliorates-PLO-and-PPD. Supplementary materials (figures, methods, tables, graphical abstract, slides, videos, Chinese translated version, and updated materials) may be found in the online DOI or iMeta Science (http://www.imeta.science).
