# Supplementary material for: Vitamin K2 ameliorates pregnancy‐ and lactation‐associated osteopenia and postpartum depression
Source: Imeta. 2026 Oct 19;5(5):e70180. doi: 10.1002/imt2.70180 (PMC13679786; doi:10.1002/imt2.70180)
Supplement: Supplementary file 1 [file IMT2-5-e70180-s001.docx]

Supporting information to

Vitamin K2 ameliorates pregnancy- and lactation-associated osteopenia and postpartum depression

Running title: Vitamin K2 ameliorates PLO and PPD via gut-bile acid signaling

Yuan, Wanqiong^1, 2, 3, #^, Jing Hang^4, 5, 6, #^, Jialin Jia^7, #^, Jie Huang^1, 2, 3^, Xuanqi Zheng^1, 2, 3^, Huan Wang^1, 2, 3^, Tong Wu^1, 2, 3^, Hao Liu^1, 2, 3^, Junxiong Zhu^8^, Chenggui Zhang^9^, Jinwen He^1, 2, 3^, Yundi Zhang^1, 2, 3^, Hong Wang^1, 2, 3^, Xin Xiong^10^, Na He^10^, Haofan Bai^11^, Jiamin Yan^4, 5, 6^, Huijie Leng^1, 2, 3^, Weili Zhu^12^, Yingsheng Xu^13, *^, Jie Shi^12, *^, Chunli Song^1, 2, 3, *^

^1^ Department of Orthopedics, Peking University Third Hospital, Beijing 100191, China.

^2^ Engineering Research Center of Bone and Joint Precision Medicine, Ministry of Education, Beijing 100191, China.

^3^ Beijing Key Laboratory of Advanced Bioadaptable Orthopedic Implants, Beijing 100191, China.

^4^ State Key Laboratory of Female Fertility Promotion, Center for Reproductive Medicine, Department of Obstetrics and Gynecology, Peking University Third Hospital, Beijing 100191, China.

^5^ National Clinical Research Center for Obstetrics and Gynecology, Key Laboratory of Assisted Reproduction, Ministry of Education, Beijing Key Laboratory of Reproductive Endocrinology and Assisted Reproduction, Beijing 100191, China.

^6^ Beijing Advanced Center of Cellular Homeostasis and Aging-Related Diseases, Institute of Advanced Clinical Medicine, Peking University, Beijing 100191, China.

^7^ Department of Orthopedics, Beijing Chaoyang Hospital, Capital Medical University, Beijing 100020, China.

^8^ Department of Orthopedics Surgery, The Second Affiliated Hospital, Zhejiang University School of Medicine, Hangzhou 310009, China.

^9^ Department of Orthopaedics, Shandong Provincial Hospital Affiliated to Shandong First Medical University, Jinan 250000, China.

^10^ Department of Pharmacy, Peking University Third Hospital, Beijing 100191, China.

^11^ School of Basic Medical Sciences, Peking University, Beijing 100038, China.

^12^ National Institute on Drug Dependence and Beijing Key Laboratory of Drug Dependence Research, Peking University, Beijing 100191, China.

^13^ Department of Neurology, Peking University Third Hospital, Beijing 100191, China.

^#^ These authors contributed equally: Wanqiong Yuan, Jing Hang, Jialin Jia.

* Corresponding author:

Chunli Song (schl@bjmu.edu.cn), Jie Shi (shijie@bjmu.edu.cn), Yingsheng Xu ([xys@bjmu.edu.cn](mailto:xys@bjmu.edu.cn))

**Supplementary Method**

**Animals and experimental design**

Twelve-week-old female nulliparous Sprague-Dawley rats (~300 g, Charles River, China), *Ocn* knockout (*Ocn* KO) mice (C57BL/6J background) and wild-type (WT) littermate controls (Shanghai Model Organisms Center, China) were housed under specific pathogen-free conditions under a 12-h dark/12-h light cycle (lights on at 8:00 am) and provided with ad libitum access to standard chow and water. Following a 7-day acclimatization period, the female rats were mated with male rats that were fed a normal diet. Upon confirmation of pregnancy, the dams were subsequently divided into five groups: the control group fed a normal diet (0.90% calcium); the PLO group fed a low-calcium diet (0.008% calcium); and the PLO + VitK2 groups received different doses (3 mg/kg, 30 mg/kg and 60 mg/kg) of VitK2 (MK-4, Sigma, V9378) daily via oral gavage. The control and PLO groups received an equivalent volume of vehicle. Diet compositions are detailed in Table S1. Each group consisted of 8 dams housed individually. After parturition, each litter was standardized to two male and two female pups (randomly selected once the sex could be reliably identified); the remaining pups were euthanized, and no cross-fostering was performed. At weaning (postnatal day 21), one male and one female pup per litter were randomly selected for all subsequent analyses (n = 8 litters per group per sex). The litter, rather than the individual pup, was considered the independent experimental unit for all offspring analyses. All offspring were weaned on Day 42 (postnatal day 21). Dams underwent behavioral testing after a one-week recovery period. The offspring rats were fed with normal diets throughout the post-weaning period, with behavioral assessments commencing at 6 weeks of age. Subsequently, animals were euthanized, and serum, bone, and fecal samples were harvested for subsequent analysis. Maternal serum indices at Day 21 and Day 42 were obtained from separate cross-sectional cohorts, and a separate cohort of age-matched female Sprague-Dawley rats from an independent batch was used for gut microbiota analyses (16S rRNA gene sequencing, n = 9-10 per group; metagenomic sequencing, n = 3 per group; Metabolomic profiling, n = 7 per group).

To investigate the role of OCN, 12-week-old female nulliparous Sprague-Dawley rats were fed a low-calcium diet and randomly divided into two groups receiving daily intraperitoneal (i.p.) injections of either PBS (vehicle control) or 4 μg/kg recombinant rat OCN protein. The His-tagged recombinant OCN was expressed in *Escherichia coli*, purified via Ni-NTA affinity chromatography, and validated by SDS-PAGE and Western blot.

Female *Ocn* KO mice aged 6-8 weeks were fed a low-calcium diet (0.008% calcium) throughout pregnancy and lactation to establish the PLO model and received either vehicle or VitK2 (60 mg/kg/day) by oral gavage. Housing, litter standardization and treatment schedules followed the Sprague-Dawley rat protocol described above. Maternal behavioral testing was performed after a one-week post-weaning recovery period, followed by bone and behavioral assessments.

This study was carried out in accordance with the National Research Council's Guide for the Care and Use of Laboratory Animals and was approved by the Ethics Committee of Biomedical Science of Peking University (LA2021553).

**Bone mineral density (BMD)**

The BMDs of the L4 vertebra and the tibia were measured by DXA using a small animal high-resolution collimator (Discovery™, Hologic Inc., USA) following previously described protocols [1].

**Bone microstructure**

The L4 vertebra and tibia were scanned by μ-CT (Inveon, Siemens, Germany) at an isotropic resolution of 8.82 μm (80 kV, 500 μA, 1500 ms exposure time). The volumes of interest were defined as follows: for the L4 vertebra, the central 2 mm of the vertebral body excluding the cortex; for the tibia, the region 1-2 mm distal to the proximal epiphyseal plate. Morphometric parameters, including bone volume/total volume (BV/TV), trabecular number (Tb.N), trabecular thickness (Tb.Th), trabecular separation (Tb.Sp), bone surface area/bone volume (BS/BV) and trabecular pattern factor (Tb.Pf) were calculated using multimodal 3D visualization software.

**Bone biomechanics**

The soft tissue was stripped from the L5 vertebra and tibia, and the mechanical properties of the bones were assessed using a mechanical testing device (Landmark, MTS, USA). Tibias were analyzed by the three-point bending test (1.8 mm/min displacement) to determine the maximum load at failure. L5 vertebrae were compressed between parallel platens (2 mm/min) until failure. The peak load, stiffness, and energy to failure were derived on the basis of the load-displacement curves generated from mechanical testing.

**High-performance liquid chromatography-tandem mass spectrometry (HPLC-MS/MS)**

The serum concentrations of VitK1, VitK2, serotonin and corticosterone were determined via HPLC-MS/MS using deuterium-labeled internal standards and multiple reaction monitoring (MRM). Samples underwent protein precipitation (formic acid for VitKs; formic acid/methanol for serotonin) followed by liquid-liquid extraction (cyclohexane for VitKs) or direct precipitation (serotonin) prior to chromatographic separation. For hippocampal deoxycholic acid (DCA) quantification, tissues were weighed and homogenized in double-distilled water (20 mg tissue/200 μL). DCA levels were determined as previously described [2].

**Sucrose preference test (SPT)**

The animals were trained to adapt to a 1% (w/v) sucrose solution for 48 h (two bottles per cage). Following 4 h of water deprivation, animals were individually housed and presented with two pre-weighed bottles (1% sucrose vs. water). After 1 h, bottles were re-weighed. Sucrose preference was calculated as: [sucrose solution consumption / (sucrose solution consumption + water consumption)] × 100%.

**Forced swim test (FST)**

For rats: Animals were placed in a Perspex cylinder (65 cm height, 24 cm diameter) filled with water (24 ± 1 °C, 45 cm depth) for a 15‑min pre‑test session. After drying, they were returned to their home cages, and a 5‑min test session was conducted 24 h later [4].

For mice: A single‑day 6‑min protocol was adopted without a separate 24‑h pre‑test session, and only the last 4 min of the session was quantified for behavioral analysis [4].

The immobility time was recorded by two independent observers blinded to group assignment. Immobility was defined as minimal movements required to keep the animal’s head above the water surface.

**Open field test (OFT)**

The OFT was performed in an apparatus (50 cm × 50 cm × 50 cm). The entry times in the center and the time spent in the center for 5 min were recorded (SMART 3.0, Panlab S.L.U., Spain). Time spent in and entries into the central zone (defined as the central 25% of the area) were quantified. The apparatus was cleaned with 75% ethanol between trials.

**Elevated plus maze (EPM)**

The apparatus consisted of four equally illuminated plastic arms (12×50×50 cm) that radiated at square angles from a central platform (12×12 cm) above the floor. Two opposite arms were enclosed by plastic walls. The other two arms were open. The maze was placed in a quiet and dimly lit room with an illumination of 100-200 lux. The animals were released from the central platform, with their faces pointing toward an enclosed arm and their behavior was recorded with a camera. Time spent in and entries into the open arms were tracked for 5 min using ANY-maze software (Stoelting Co., USA). An entry was defined as the center of mass of the animal entering the arm.

**Morris water maze (MWM)**

The MWM test was conducted in a round black pool 150 cm in diameter. The pool was filled to a depth of 35 cm with water (24 °C). The escape platform was placed in the center of one quadrant of the pool, which was equally divided into four quadrants. Before being placed in the water, the animal was allowed to stand on the platform for 2 min to become familiar with the hidden platform and to memorize the environment. The animal was then placed into the water facing the mid-section of the wall in one of the four quadrants and allowed to swim freely until it found and climbed onto the platform. If the animals failed to locate the platform within 2 min, they were guided to it and allowed to remain for 15 s. Spatial training was continuously conducted for 7 days.

**Novel object recognition (NOR)**

The test comprised a familiarization phase (10 min exposure to two identical objects) and a test phase 24 h later (10 min exposure to one familiar and one novel object). Familiarization phase: The animals were individually placed in an open-field arena (40 cm × 40 cm × 30 cm) containing two identical objects. The objects were placed in the arena at the same time, and the rats were allowed to freely explore the objects. Test phase: The animals were returned to the same arena. One of the familiar objects was replaced by a novel object, and the animals were allowed to explore both objects. The exploration time for each object was recorded. The discrimination index (DI) was calculated. DI = (Time_novel object - Time spent_familiar object) / (Time_novel object + Time spent_familiar object).

**Electrophysiology**

**Action potential**

Current-clamp recordings were performed using a MultiClamp 700B amplifier (Molecular Devices). Glass pipettes with resistances of 3-5 MΩ were filled with a K⁺-based internal solution containing (in mM): 130 potassium methanesulfonate, 10 KCl, 10 HEPES, 0.4 EGTA, 2 MgCl₂, 2.5 Mg-ATP, and 0.25 Na₃GTP. Recordings with a series resistance of < 30 MΩ were accepted. The recorded neurons were stimulated with 1-s depolarizing current steps starting at 0 pA and increasing in 20-pA increments to a maximum of +200 pA, with an interstimulus interval of 15 s. The firing frequency was calculated by dividing the number of action potentials evoked during each current step by the 1-s stimulus duration and was plotted against the injected current to generate the frequency-current relationship.

**Spontaneous excitatory postsynaptic currents (sEPSCs)**

Recordings were obtained in voltage-clamp mode. Patch pipettes were filled with a cesium-based internal solution containing the following solutions (in mM): 135 cesium methanesulfonate, 10 KCl, 1 MgCl_2_, 0.2 EGTA, 2 QX-314, 4 ATP-Mg, 0.3 GTP-Na, and 20 phosphocreatine (pH 7.3, 290-295 mOsm). The holding potential was maintained at −70 mV, and the series resistance was routinely monitored. Bicuculline (20 μM) was added to ACSF to block gamma-aminobutyric acid type A receptors throughout the recording process, and each cell was recorded for 5 min. For analysis, postsynaptic events were detected by Clampfit 10.7 software (Molecular Devices).

**Long-term potentiation recordings (LTP)**

Whole-cell voltage-clamp recordings were performed using the same cesium-based internal solution as that used for sEPSC recordings. Evoked excitatory postsynaptic currents (eEPSCs) were recorded from hippocampal CA1 pyramidal neurons following stimulation of the Schaffer collateral pathway with a concentric bipolar stimulating electrode (FHC, Bowdoin, ME, USA). After a stable baseline was established, LTP was induced using a single 0.5-s train of 50 pulses delivered at 100 Hz. eEPSCs were subsequently recorded for 60 min. LTP magnitude was quantified as the mean eEPSC amplitude during the final 10 min of recording and expressed as a percentage of the baseline amplitude.

**16S rRNA sequencing**

Microbial genomic DNA was extracted from feces (MagPure Soil DNA KF Kit). The genomic DNA was amplified in 50 μL triplicate reactions with bacterial 16S rRNA gene (V3-V4 region)-specific primers: 343F (5’-TACGGRAGGCAGCAG-3’) and 798R (5’-AGGGTATCTAATCCT-3’). The reverse primer incorporated a unique sample barcode, with both amplification primers linked to Illumina sequencing adapters. Following purification, the PCR product concentrations were standardized to facilitate sequencing on the Illumina NovaSeq 6000 platform to generate 250-bp paired-end reads.

Raw sequencing reads were demultiplexed by sample barcode, and primer and adapter sequences were removed using Cutadapt. Reads were quality-filtered, denoised, merged and screened for chimeras using DADA2 in QIIME 2 (2020.11). Representative sequences and an amplicon sequence variant (ASV) abundance table were generated. Taxonomic assignments were obtained against SILVA (version 138) using q2-feature-classifier with default settings. Alpha diversity was assessed using ACE, Faith’s phylogenetic diversity (PD whole tree) and Shannon indices. PCA was used to visualize between-sample variation in the microbial abundance profiles (Figure S7D).

Differential taxa were screened using LEfSe with an LDA score threshold of 4.0. The core taxa, *Bacteroidaceae* and *Bacteroides*, were additionally assessed using linear models of log_10_-transformed, total-sum-scaled relative abundances. *P* values were adjusted using the Benjamini-Hochberg (BH) procedure, with *q* < 0.05 indicating statistical significance. Microbial functions were predicted using PICRUSt2 (version 2.3.0b0), Pathway comparisons used FDR-adjusted *p*-values. Within the 16S experiment, all samples were collected, processed and sequenced in a single batch.

**Metagenomic sequencing**

**DNA isolation and library construction**

Total DNA was isolated from samples using a QIAamp^®^ Fast DNA Stool Mini Kit (Qiagen, Germany). The DNA was fragmented with S220 focused ultrasonicators (Covaris, USA) and cleaned with Agencourt AMPure XP beads (Beckman Coulter Co., USA). The libraries were subsequently constructed using a TruSeq Nano DNA LT Sample Preparation Kit (Illumina, USA).

**Bioinformatic analysis**

Libraries were sequenced on an Illumina NovaSeq 6000 platform to generate 150-bp paired-end reads. Trimmomatic (version 0.36) was used for adapter trimming and quality filtering. Filtered reads were aligned to the *Rattus norvegicus* reference genome (Rnor_6.0; Ensembl release 104) using Bowtie2 (version 2.2.9), and host-mapped reads were discarded. The remaining reads were assembled using MEGAHIT (version 1.1.2). Scaffolds were split at gaps, and contigs longer than 500 bp were retained. Open reading frames were predicted using Prodigal (version 2.6.3) and translated into amino acid sequences. CD-HIT (version 4.5.7) was used to construct a nonredundant gene catalog at 95% sequence identity and 90% coverage. The longest sequence in each cluster was retained as its representative.

Gene abundance quantification was performed by Bowtie2 v2.2.9 alignment against the nonredundant catalog (95% identity). Taxonomic annotation was performed with reference to the NCBI NR database (release version 2022.03), and species abundance was derived from gene abundance profiles. Hierarchical abundance statistics spanned eight taxonomic ranks (domain-species). The functional annotation employed DIAMOND v0.9.7 and the KEGG (Release 98.1) and GO (2022-01 releases) databases (E value ≤ 1×10^-5^).

Alpha diversity was assessed using the ACE and Shannon indices. PCA was performed in R (version 4.2.1) to summarize variation among metagenomic abundance profiles. Intergroup differences were assessed using the Kruskal-Wallis test in R (v4.2.1). Species-level relative abundance was derived from the annotated gene-abundance profiles. Bacteroides-related species abundances were displayed as log_10_(relative abundance × 100). KEGG functional profiles were summarized at the pathway level. Within the metagenomic experiment, all samples were collected, processed and sequenced in a single batch.

**Metabolomic profiling**

**Sample Preparation**

The samples stored at -80 °C were thawed at room temperature. A total of 100 μL of sample was mixed with 300 μL of ice-cold methanol/acetonitrile (2:1, v/v) containing L-2-chlorophenylalanine (0.06 mg/mL). Samples were vortexed for 1 min, sonicated in an ice-water bath for 10 min and incubated at −40 °C for 2 h. Extracts were centrifuged at 13,000 rpm for 10 min at 4 °C. A 150-μL supernatant aliquot was transferred to a glass vial for subsequent platform-specific sample preparation. Pooled QC samples were prepared by combining equal aliquots of the study-sample extracts. Within each metabolomics platform, sample preparation and analysis were performed in a single batch, with study samples injected in a randomized order. Pooled QC samples were injected at regular intervals to monitor analytical reproducibility. Features with poor reproducibility in QC samples were excluded according to the project-specific criteria.

**GC-MS analysis**

The supernatant aliquot was vacuum-dried at room temperature. The residue was mixed with methoxyamine hydrochloride in pyridine (15 mg/mL). Samples were vortexed for 2 min and incubated at 37 °C for 90 min. BSTFA containing 1% TMCS and n-hexane were then added. Samples were vortexed for 2 min, derivatized at 70 °C for 60 min and equilibrated at room temperature for 30 min before GC-MS analysis.

Serum metabolites were analyzed using an Agilent 7890B GC-5977A MSD (Agilent Technologies Inc., CA, USA) with a DB-5MS column (30 m × 0.25 mm × 0.25 μm). The helium ( > 99.999%) carrier gas flow rate was 1 mL/min under splitless injection (260 °C). The temperature gradient was as follows: 60 °C (0.5 min)→125 °C at 8 °C/min→210 °C at 5 °C/min→270 °C at 10 °C/min→305 °C at 20 °C/min (5 min hold). The MS instrument was operated in EI mode (70 eV) with a quadrupole of 150 °C, an ion source of 230 °C, and scanning m/z 50-500.

Raw GC-MS .D files were converted to .abf format using Analysis Base File Converter and processed with MS-DIAL. Processing included peak detection, spectral deconvolution, peak alignment and missing-value interpolation. Metabolite annotations were assigned using the LUG database. Peak intensities were normalized to screened internal-standard signals, followed by redundant-feature removal and peak merging to generate the final data matrix.

PCA was performed in R to visualize the GC-MS metabolomic profiles. Pairwise differences in metabolite intensities were assessed using Kruskal-Wallis. *P* values were adjusted using the BH procedure across the complete tested metabolite-feature set for each comparison. Differential metabolites were defined by VIP > 1.0 and BH-adjusted *q* < 0.05 and were used for KEGG pathway enrichment analysis. VIP scores were obtained from the OPLS-DA model

**LC-MS/MS analysis**

For LC-MS analysis, the supernatant aliquot was dried in a centrifugal concentrator and reconstituted in methanol/water (1:4, v/v). Samples were vortexed for 30 s, sonicated in an ice-water bath for 3 min and incubated at -20 °C for 2 h. After centrifugation at 13,000 rpm for 10 min at 4 °C, the supernatant was filtered through a 0.22-μm filter. The filtrate was transferred to an LC vial and stored at -80 °C until analysis.

Serum metabolites were analyzed using an ACQUITY UPLC I-Class Plus (Waters) coupled to a Q Exactive MS (Thermo Fisher) with a HESI source in positive/negative mode. Chromatographic separation was performed with an HSS T3 column (2.1×100 mm, 1.8 μm) at 45 °C with a 0.35 mL/min gradient: 5% B (0.01-2 min)→30% B at 4 min→50% B at 8 min→80% B at 10 min→100% B (14-15 min)→5% B (15.1-16 min). Mobile phase: (A) 0.1% formic acid-water mixture; (B) 0.1% formic acid-acetonitrile mixture. MS parameters: m/z 100-1,000 scan range; 70,000 (MS1)/17,500 (MS2) resolution; 10/20/40 eV stepped HCD; spray voltage 3.8 kV (+)/3.2 kV (-); sheath gas 35 arb; capillary 320 °C.

Raw LC-MS data were processed using Progenesis QI (version 2.3) for baseline filtering, peak detection and integration, retention-time correction, peak alignment and normalization. Precursor and product-ion tolerances were 5 and 10 ppm, respectively, with a product-ion threshold of 5%. Metabolites were annotated using accurate mass, MS/MS fragments and isotope patterns against HMDB, LIPID MAPS (version 2.3), METLIN and an in-house database. Features with matching scores below 36 out of 60 were excluded. Features with zero intensity in more than 50% of samples within a group were removed. Remaining zero values were replaced with half the minimum value, and positive- and negative-ion data were combined into a data matrix.

PCA was performed in R to visualize LC-MS metabolomic profiles. Pairwise differences were assessed using Kruskal-Wallis, with BH correction across the complete tested metabolite-feature set for each comparison. Differential metabolites were defined by VIP > 1.0 and BH-adjusted *q* < 0.05 and were subjected to KEGG pathway enrichment analysis. VIP scores were obtained from the OPLS-DA model.

**Combined analysis of LC-MS/MS and GC-MS data**

We integrated the LC-MS/MS positive- and negative-ion data with the GC-MS data. For metabolites detected by both analytical platforms, only those with higher qualitative scores were retained. This consolidated dataset encompasses all analyzable information extracted from the raw data and was used as the primary basis for all subsequent analyses.

Associations between microbial features, metabolites and phenotypic measures were assessed using Spearman’s rank correlation. Correlation P values were adjusted using the BH procedure. Adjusted *q* values were used to determine statistical significance.

**Cell culture and treatment**

The MC3T3-E1 cells (CVCL_0409) and RAW264.7 cells (CVCL_0493) were obtained from the Cell Resource Center, Institute of Basic Medical Science (Beijing, China). SH-SY5Y cells (CRL-2266) were obtained from the American Type Culture Collection (ATCC). MC3T3-E1 cells were cultured in α-MEM (Gibco, USA), RAW264.7 cells were cultured in DMEM (Gibco, USA), and SH-SY5Y cells were cultured in a 1:1 mixture of ATCC-formulated Eagle's Minimum Essential Medium (EMEM) and F12 Medium. All these cells were supplemented with 10% FBS and 1% penicillin-streptomycin.

Cells were seeded at a density of 5 × 10⁵ cells/mL. Differentiation day 0 was defined as the day on which the growth medium was replaced with differentiation medium. Upon reaching approximately 80% confluence, MC3T3-E1 cells were cultured in osteogenic differentiation medium (OriCell, MUXMX-90021). RAW264.7 cells were cultured in differentiation medium containing 50 ng/mL M-CSF (PeproTech, 315-02) and 50 ng/mL RANKL (PeproTech, 315-11). Both cell types were differentiated for 7 days. MC3T3-E1 cells were assessed by ALP staining and quantitative real-time PCR for *Runx2* and *Alp* on day 7. RAW264.7 cells were assessed by TRAP staining and real-time PCR for *Nfatc1* on day 7.

For neuronal differentiation, SH-SY5Y cells were cultured in EMEM/F-12 supplemented with 2% FBS and 10 μM all-trans retinoic acid (RA; Sigma-Aldrich, R2625) for 3 days. Cells were then maintained in RA-containing differentiation medium supplemented with 50 ng/mL brain-derived neurotrophic factor (BDNF; PeproTech, 450-02) for an additional 7 days. *GluA1* expression was measured by real-time PCR at the end of this induction period on day 10. For GABAergic differentiation, cells underwent the same initial 10-day induction, followed by an additional 3 days in differentiation medium containing 1 mM dibutyryl cyclic AMP (dbcAMP; Sigma-Aldrich, D0260). *Gat1*, *Gad65* and *Gad67* expression was measured by qPCR on day 13.

For all differentiation experiments, DCA (Sigma-Aldrich, 30960) was added at 0, 50 or 100 μM from day 0 until the corresponding differentiation endpoint. Differentiation medium was replaced every 3 days, with DCA replenished at the assigned concentration at each medium change. Thus, DCA exposure lasted 7 days for MC3T3-E1 and RAW264.7 cells, 10 days for the RA/BDNF-treated SH-SY5Y cells, and 13 days for the GABAergic induction condition.

**CCK-8 assay**

SH-SY5Y cells were seeded into 96-well plates at a density of 5 × 10³ cells per well in 100 μL of complete medium. After 24 h of attachment, cells were treated with various concentrations of DCA (0, 50, 100 μM). At each time point, 10 μL of CCK-8 solution (Dojindo, CK04) was added to each well, and the plates were incubated at 37 °C for 2 h. Absorbance at 450 nm (OD450) was measured using a microplate reader. All experiments were performed in triplicate.

**Alkaline phosphatase (ALP) staining**

The cells were fixed with 4% paraformaldehyde and the ALP staining was performed according to the manufacturer's instructions (Applygen, E1043).

**Tartrate-resistant acid phosphatase (TRAP) staining**

The cells were fixed with 4% paraformaldehyde and TRAP staining was performed according to the manufacturer's instructions (Solarbio, G1492).

**Quantitative real-time PCR (qPCR)**

Total RNA was extracted from lumbar and brain tissues with TRIzol reagent (Life Technologies, 15596018) and reverse transcribed into cDNA with a PrimeScript RT Reagent Kit (Takara, PR037A). Real-time PCR was performed with SYBR Green PCR Master Mix (Toyobo, QPK-201). The primers used are listed in Table S2. Data from each sample were identified by melting curve analysis and normalized to GAPDH, and every experiment was performed at least three times. The relative expression levels of each target gene were calculated using the 2-(△△Ct) method.

**Western blot**

Western blot was performed as previously described [1]. The signals were detected with an iBright CL1000 Imaging System (Thermo Fisher, USA). For tryptophan hydroxylase (TPH) (Abcam, ab217024), glutamic acid decarboxylase 67 (GAD67) (Abcam, ab26116), p-GluA1 (Ser845) (CST, 8084), GluA1 (Abcam, ab109450), His (CST, 12698) and GAPDH (CST, 2118) detection, 40 μg of total protein from each sample was loaded.

**TRAP staining**

TRAP staining of the tibia was performed using a commercial kit (Servicebio, G1050) in accordance with the manufacturer’s instructions.

**Masson's trichrome staining**

The tibia was fixed, dehydrated, embedded, sectioned, and subjected to Masson’s trichrome staining in accordance with the manufacturer’s instructions (Servicebio, G1006)**.**

**Immunohistochemistry (IHC)**

IHC was performed on formalin-fixed, paraffin-embedded tissues from animals as previously described [3]. An antibody against Doublecortin (DCX) (Abcam, AB18723) was used as the primary antibody for IHC.

**Nissl staining**

Nissl staining was performed as previously described [4].

**Enzyme-linked immunosorbent assay (ELISA)**

‌PTH (JonlnBio, JL21082), PTHrP (JonlnBio, JL11145), OCN (Meimian, MM-20603R1), Gla-OCN (Takara, EIA kits MK126) and Glu-OCN (Takara, EIA kits MK146) levels were analyzed according to the manufacturer's instructions.

**Statistical analysis**

Statistical analysis was performed using GraphPad Prism 10.0 software. All data are presented as mean ± standard deviation (SD), with significance defined as *p* < 0.05. For differences between two groups, two-tailed unpaired Student's t-tests were used. For comparisons among more than two groups, two-way ANOVA was utilized. MWM tests were analyzed separately using a two-way repeated-measures ANOVA, with treatment group as the between-subject factor and training day as the within-subject factor. Greenhouse-Geisser correction was applied to the day and group-by-day interaction effects. Overall group contrasts across the seven training days were adjusted using the Holm procedure across eight comparisons spanning both sexes. Action potential firing frequency-current relationships were analyzed using two-way repeated-measures ANOVA, followed by Tukey’s multiple-comparisons test. sEPSC amplitude and frequency, as well as eEPSC potentiation during the final 10 min of LTP recordings, were analyzed using mixed-effects models to account for multiple cells or slices obtained from the same animal, followed by Tukey’s multiple-comparisons test. Correlations were assessed using Spearman’s rank correlation test and adjusted for multiple comparisons using the Benjamini-Hochberg procedure. In the figures, *p* values are denoted as follows: ns, not significant; * or #, *p* < 0.05; ** or ##, *p* < 0.01; *** or ###, *p* < 0.001.

**References**

1.	Fan, Dongwei, Daoyang Fan, Wanqiong Yuan. 2021. "CMTM3 suppresses bone formation and osteogenic differentiation of mesenchymal stem cells through inhibiting Erk1/2 and RUNX2 pathways." *Genes & Diseases* 8: 882−890. <http://dx.doi.org/10.1016/j.gendis.2020.12.003>

2.	Li, Chenli, Liangjie Wang, Wenting Xie, Erqu Chen, Yanbing Chen, Huifang Li, Dan Can, et al. 2024. "TGR5 deficiency in excitatory neurons ameliorates Alzheimer's pathology by regulating APP processing." *Science Advances* 10: eado1855. <http://dx.doi.org/10.1126/sciadv.ado1855>

3.	Shi, Tianshu, Siyu Shen, Yong Shi, Qianjin Wang, Guanqun Zhang, Jiaquan Lin, Jiang Chen, et al. 2024. "Osteocyte-derived sclerostin impairs cognitive function during ageing and Alzheimer's disease progression." *Nature Metabolism* 6: 531−549. <http://dx.doi.org/10.1038/s42255-024-00989-x>

4.	Wu, Jialin, Huachong Xu, Shiqi Wang, Huandi Weng, Zhihua Luo, Guosen Ou, Yaokang Chen, et al. 2024. "Regular exercise ameliorates high-fat diet-induced depressive-like behaviors by activating hippocampal neuronal autophagy and enhancing synaptic plasticity." *Cell Death & Disease* 15: 737. <http://dx.doi.org/10.1038/s41419-024-07132-4>

**Supplementary figures**

Figure S1 Low-calcium diet induces pregnancy- and lactation-associated osteopenia (PLO) in maternal rats. (A) Schematic illustration of the experimental design for the PLO model. (B) Serum calcium levels measured by the Arsenazo III method in maternal rats. (C) Representative micro-computed tomography (micro-CT) images of the L4 vertebra (top) and tibia (bottom) in control and low-calcium diet rats. (D-G) Quantitative analysis of maternal trabecular bone microarchitecture parameters: bone volume fraction (BV/TV), (D) trabecular number (Tb.N) (E), trabecular thickness (Tb.Th) (F), and trabecular separation (Tb.Sp) (G). (H) Bone mineral density (BMD) of the lumbar spine and tibia assessed by dual-energy X-ray absorptiometry (DXA). (I-K) Biomechanical properties, including peak load (I), stiffness (J), and energy to failure (K) of the L5 vertebra and tibia determined by mechanical testing. This experiment used an animal cohort independent of that used for the VitK2 intervention experiment in Figure 1. n = 6 rats per group. *, *p* < 0.05, **, *p* < 0.01, ***, *p* < 0.001.

## Figure S2 Serum vitamin K levels under different physiological and dietary conditions. All serum Vitamin K levels were measured by high-performance liquid chromatography (HPLC). (A) Vitamin K2 (VitK2) levels in nulliparous and parous rats at the end of pregnancy (Day 21) and lactation (Day 42) with normal diets (n = 7). (B) VitK2 levels in nulliparous rats with normal (control) or low-calcium diets on Day 21 and Day 42 (n = 9). (C) Vitamin K1 (VitK1) levels in nulliparous and parous rats fed a normal diet (n = 7). (D) VitK1 levels in nulliparous rats with a control or low-calcium diet (n = 6). (E) VitK1 levels in maternal rats fed a control diet, a low-calcium diet, or a low-calcium diet supplemented with different doses of VitK2 at the end of pregnancy and lactation (n = 7-8). PLO: pregnancy- and lactation-associated osteopenia, ns: not significant, *: vs. Control, #: vs. PLO. *: *p* < 0.05, ** or ##: *p* < 0.01, *** or ###: *p* < 0.001.

**Figure S3 Effects of VitK2 on calcium-regulating hormones and bone properties in PLO rats.** (A) Body weight of maternal rats. (B) Serum calcium levels measured by the Arsenazo III method at the end of pregnancy (Day21) and lactation (Day42). (C) Serum PTH levels measured by enzyme-linked immunosorbent assay (ELISA). (D) Serum PTHrP levels measured by ELISA. (E) Quantitative micro-CT analysis of L4 vertebral trabecular pattern factor (Tb.Pf). (F) Energy to failure of the lumbar spine analyzed by MTS Landmark Systems. Panels A and C-F were performed at one week after offspring weaning. n = 7-8 rats per group. PLO: pregnancy- and lactation-associated osteopenia, *: vs. Control, #: vs. PLO. * or #: *p* < 0.05, ** or ##: *p* < 0.01, *** or ###: *p* < 0.001.

**Figure S4 Maternal serum serotonin levels during pregnancy and lactation.** Serum serotonin levels at the end of pregnancy (Day 21) and lactation (Day 42) were measured by HPLC. n = 8 rats per group. PLO: pregnancy- and lactation-associated osteopenia, *: vs. Control, #: vs. PLO. * or #: *p* < 0.05, **: *p* < 0.01, ***: *p* < 0.001.

## Figure S5 Serum corticosterone levels. Serum corticosterone in maternal rats fed a control diet, a low-calcium diet, or a low-calcium diet supplemented with different doses of VitK2 at the end of lactation by HPLC (Day 42). n = 7-8 per group. PLO: pregnancy- and lactation-associated osteopenia. *: *p* < 0.05.

**Figure S6 VitK2 ameliorated low-calcium diet-induced PLO and PPD independently of osteocalcin (OCN).** (A-C) Total OCN (A), Gla-OCN (B), and Glu-OCN (C) levels in maternal rats measured by ELISA. (D-F) Total OCN (D), Gla-OCN (E) and Glu-OCN (F) levels in male offspring measured by ELISA. In A-F, Control denotes normal-diet maternal rats or their offspring, whereas PLO denotes low-calcium-fed maternal rats or their offspring. (G) Validation of recombinant rat OCN protein by SDS-PAGE. (H) Western blot detection of His-tagged recombinant OCN using an anti-his antibody. (I-P) Rescue experiments in maternal rats that received intraperitoneal injections of recombinant OCN (4 μg/kg daily, intraperitoneally): serum Gla-OCN (I) and serum Glu-OCN (J) levels measured by ELISA; BMD of tibia (K); Masson's trichrome staining of tibia (Scale bar: 500 μm) (L); TRAP staining of tibia (Scale bar: 500 μm) (M); Sucrose preference test (SPT) (N); percentage of time spent in the open arms of the Elevated plus maze test (EPM) (O); and Morris water maze test (P). In I-P, Control denotes the low-calcium + PBS group. (Q-T) Experiments in *Ocn* knockout (KO) mice: Open field test in wild-type (WT) and *Ocn* KO mice (Q-R); EPM test in WT and *Ocn* KO mice (S-T). (U, V) BMD of tibias (U) and sucrose preference (V) in *Ocn*-KO dams with PLO induced by a low-calcium diet during pregnancy and lactation, treated with vehicle or VitK2 (60 mg/kg daily). For panels U and V, n = 6 mice per group; for all other applicable panels, n = 7 animals per group. PLO: pregnancy- and lactation-associated osteopenia. *: vs. Control, #: vs. PLO. * or #: *p* < 0.05, **: *p* < 0.01, ***: *p* < 0.001.

Figure S7 16S rRNA gene sequencing analysis of the maternal rats. (A-C) 16S rRNA gene sequencing analysis. Alpha diversity is represented by ACE (A), PD-whole-tree (B), and Shannon (C) indices. (D) Principal component analysis (PCA) of microbial community profiles. Overall differences among the five groups were assessed by permutational multivariate analysis of variance (PERMANOVA; P = 0.001). (E) KEGG pathway analysis of enriched biological processes. (F and G) Heatmaps of differential taxa at the family (F) and genus (G) levels. (H) Random forest classifier importance plot. (I) Relative abundance of the family *Bacteroidaceae*. (J and K) Spearman’s correlation analysis between bone/depressive-like behavioral indices and differential taxa at the family (J) and genus (K) levels, respectively. *: *p* < 0.05, **: *p* < 0.01, ***: *p* < 0.001. PLO: pregnancy- and lactation-associated osteopenia. n = 9-10 rats per group.

Figure S8 Metagenomic profiling of the gut microbiota in maternal rats. (A-C) Alpha diversity was represented by ACE (A) and Shannon (B) indices, and Principal Component Analysis (PCA) of sample distribution (C). (D) Relative abundances of the displayed *Bacteroides*-related species, plotted as log_10_(relative abundance × 100). (E) Heatmap of KEGG Orthology (KO) analysis of the metagenomic data, including secondary bile acid biosynthesis (ko00121). (F) Relative abundance of the species *Clostridium scindens* (*C. scindens*) by metagenomic sequencing. PLO: pregnancy- and lactation-associated osteopenia. n = 3 rats per group. *: *p* < 0.05.

**Figure S9 Multi-omics analysis of the maternal rats.** (A) PCA plot of serum metabolomic profiling based on GC-MS data. (B) PCA plot of serum metabolomic profiling based on LC-MS data. (C) Venn diagram showing differential metabolites identified in pairwise comparisons. (D) Heatmap showing the 17 metabolites exhibiting significant abundance differences between Control vs. PLO and PLO vs. PLO + VitK2 groups, but not between Control vs. PLO + VitK2 groups. (E) Heatmaps showing metabolites exhibiting a V-shaped trend. (F) Correlation analysis between the above V-shaped metabolites and bone/depressive-like behavioral indices. (G) Heatmap of the bile acid-related metabolites. (H) Bubble plot of KEGG pathway enrichment for all the differential metabolites across the Control, PLO, and PLO+VitK2 groups. (I) Correlation network between *Bacteroides* and DCA. (J) Correlation analysis between *Bacteroides* and DCA. (K) Correlation network between *B. uniformis* and DCA. (L) Bipartite network constructed from KEGG Markup Language (KGML) analysis linking fecal metagenomic and serum metabolomic data. *: *p* < 0.05, **: *p* < 0.01, ***: *p* < 0.001. PLO: pregnancy- and lactation-associated osteopenia.

**Figure S10 Multi-omics sequencing reveals that VitK2 ameliorates postpartum depression.** (A) Box plot of metagenomic gene counts per group. (B) Venn diagram showing shared gene sets. (C) Relative abundance of differentially enriched KEGG functional modules based on metagenomic data. M00020: Serine biosynthesis, glycerate-3P => serine. M00077: Chondroitin sulfate degradation. M00027: GABA (gamma-Aminobutyrate) shunt. M00882: Lipoic acid biosynthesis, eukaryotes, octanoyl-ACP => dihydrolipoyl-H. M00065: GPI-anchor biosynthesis, core oligosaccharide. M00970: Proline degradation, proline => glutamate. M00155: Cytochrome c oxidase, prokaryotes. M00894: Cannabidiol biosynthesis, malonyl-CoA => cannabidiol/dronabinol. (D) Relative abundance of glutaminase (GLS) and glutamate decarboxylase (GAD) genes derived from metagenomic sequencing data. (E) The intensities of acetylcholine in the hippocampus by targeted metabolomics. (F) The intensities of taurine in the hippocampus by targeted metabolomics. (G) The expression of *tryptophan hydroxylase 2* (*Tph2*), glutamate decarboxylase *Gad1* (encoding GAD67) and *Gad2* (encoding GAD65) in the brain of maternal rats by real-time PCR. (H) Western blot of GAD67 and TPH1 in the brain of maternal rats. (I) Western blot of hippocampal p-glutamate receptor 1 (GluA1) (Ser845) and total GluA1 expression. (J) Correlation analysis of TPH1, GAD67, p-GluA1 (Ser845), and GluA1 expression with behavioral indicators. (K) Cell counting kit-8 (CCK-8) assay of SH-SY5Y cells treated with different doses of DCA. For panels A-H, n = 3 biological replicates per group. *: vs. Control, #: vs. PLO. * or #: *p* < 0.05, **: *p* < 0.01, ***: *p* < 0.001. PLO: pregnancy- and lactation-associated osteopenia.

**Figure S11 Maternal VitK2 supplementation improves female offspring cognition.** (A) Serum VitK2 levels in offspring nursed by maternal rats treated with different doses of VitK2. (B) Serum calcium levels measured by the Arsenazo III method in offspring rats. (C) Representative micro-CT images of the L4 vertebra in offspring rats. (D-I) Quantitative micro-CT parameters in offspring rats: BV/TV (D), Tb.N (E), Tb.Th (F), Tb.Sp (G), BS/BV (H), and Tb.Pf (I). (J) BMD of the lumbar spine was analyzed by DXA in offspring rats. (K) Peak load of the lumbar spine. (L) Representative routes from the Morris water maze test (MWM). (M) The mean escape latency time of the MWM test. (N) Discrimination index in the Novel Object Recognition test. (O) Representative traces of evoked EPSC (eEPSC) before (left) and 60 min after high-frequency stimulation (HFS; middle), and overlaid traces (right). (P) The average eEPSC plotted against time in minutes of the offspring rats (12-13 slices, n = 4-5 rats per group). (Q) The average eEPSC peak amplitude over the last 10 minutes of recording, normalized to baseline. (R) Representative images of hippocampal neurons by Nissl's staining. (**S**) Doublecortin (DCX)-positive cells in the hippocampus via immunohistochemistry. n represents the number of litters; one pup per sex per litter was analyzed. For panels A-N, 6 female offspring from 6 independent litters per group. PLO: pregnancy- and lactation-associated osteopenia. *: vs. Control, #: vs. PLO. * or #: *p* < 0.05, **or ##: *p* < 0.01, ***or ###: *p* < 0.01.

**Figure S12 Maternal VitK2 supplementation improves cognitive function in male offspring.** (A) Serum VitK2 levels in male offspring that were lactated by maternal rats with different doses of VitK2. (B) Serum calcium levels measured by the Arsenazo III method in offspring. (C-F) Quantitative micro-CT parameters in male offspring rats: BV/TV (C), Tb.N (D), Tb.Th (E) and Tb.Sp (F). (G) Representative routes from the Morris water maze (MWM) test. (H) The mean escape latency time of male offspring during the MWM test. (I) Discrimination index in the Novel Object Recognition (NOR) test of male offspring. n represents the number of litters; one pup per sex per litter was analyzed. n = 6-8 rats per group. PLO: pregnancy- and lactation-associated osteopenia. *: vs. Control, #: vs. PLO. * or #: *p* < 0.05, ** or ##: *p* < 0.01, *** or ###: *p* < 0.001.

**Figure S13 VitK2 alleviates depressive-like behaviors in nulliparous mice fed with a low-calcium diet.** (A) Serum serotonin levels in nulliparous and parous C57BL/6J mice fed a normal diet at the end of pregnancy (Day 21) and lactation (Day 42) measured by HPLC. (B) Sucrose preference test (SPT). (C) Forced swim test. Control: normal diets. Low calcium: low-calcium-diets. Low calcium + VitK2: low-calcium-diets with VitK2 supplementation (VitK2: 60 mg/kg daily for 42 days). n = 6-8 per group. *: vs. Control, #: vs. Low calcium. * or #: *p* < 0.05.
